# *P. falciparum* Enhances HIV Replication in an Experimental Malaria Challenge System

**DOI:** 10.1371/journal.pone.0039000

**Published:** 2012-06-26

**Authors:** Marika Orlov, Florin Vaida, Olivia C. Finney, David M. Smith, Angela K. Talley, Ruobing Wang, Stefan H. Kappe, Qianqian Deng, Robert T. Schooley, Patrick E. Duffy

**Affiliations:** 1 Seattle Biomedical Research Institute, Seattle, Washington, United States of America; 2 Department of Medicine, University of California San Diego, La Jolla, California, United States of America; 3 Department of Family and Preventive Medicine, University of California San Diego, La Jolla, California, United States of America; 4 Laboratory of Malaria Immunology, National Institute of Allergy and Infectious Diseases (NIAID), National Institutes of Health (NIH), Rockville, Maryland, United States of America; Burnet Institute, Australia

## Abstract

Co-infection with HIV and *P. falciparum* worsens the prognosis of both infections; however, the mechanisms driving this adverse interaction are not fully delineated. To evaluate this, we studied HIV-1 and *P. falciparum* interactions *in vitro* using peripheral blood mononuclear cells (PBMCs) from human malaria naïve volunteers experimentally infected with *P. falciparum* in a malaria challenge trial.PBMCs collected before the malaria challenge and at several time points post-infection were infected with HIV-1 and co-cultured with either *P. falciparum* infected (iRBCs) or uninfected (uRBCs) red blood cells. HIV p24Ag and TNF-α, IFN-γ, IL-4, IL-6, IL-10, IL-17, and MIP-1α were quantified in the co-culture supernatants. In general, iRBCs stimulated more HIV p24Ag production by PBMCs than did uRBCs. HIV p24Ag production by PBMCs in the presence of iRBCs (but not uRBCs) further increased during convalescence (days 35, 56, and 90 post-challenge). In parallel, iRBCs induced higher secretion of pro-inflammatory cytokines (TNF-α, IFN-γ, and MIP-1α) than uRBCs, and production increased further during convalescence. Because the increase in p24Ag production occurred after parasitemia and generalized immune activation had resolved, our results suggest that enhanced HIV production is related to the development of anti-malaria immunity and may be mediated by pro-inflammatory cytokines.

## Introduction

HIV-1 and *Plasmodium falciparum* malaria remain two of Sub-Saharan Africa’s major causes of morbidity and mortality. Together malaria and HIV caused nearly 2.5 million deaths in Africa during 2008. Although it was not initially fully appreciated [1], [2], increasing evidence indicates that the two pathogens interact in individuals and in populations. During acute bouts of clinical malaria, plasma HIV-1 RNA levels rise [3]–[5], and CD4 cells decline by approximately 40 cells/µL/year with each malaria episode [6] compared to the rate of decline in individuals without clinical malaria episodes. Conversely, in regions of unstable malaria transmission, HIV infection is associated with increased malaria disease severity and death [7]. Recently, a mathematical model further explored the potential importance of the malaria/HIV interaction. Based on this model, in an area of Kenya, with an adult population of roughly 200,000 that has been exposed to both pathogens since 1980, the interaction of the two diseases may have caused 8,500 excess HIV infections and 980,000 excess malaria episodes [8]. Supporting this mathematical model, a study that examined geographical overlap of the two pathogens in East Africa found that those who live in areas of high *P. falciparum* incidence have about twice the risk of being HIV infected compared to individuals who live in areas of low incidence [9].

The host immune response to *Plasmodium* infection includes the rapid release of interferon-gamma (IFN-γ) by natural killer (NK) cells and tumor necrosis factor (TNF) by macrophages, while cells of the adaptive immune system release TNF, interleukin (IL)-12, and IFN-γ in response to parasitized erythrocytes [10]. Several cytokines that are elaborated during acute bouts of malaria can cause stimulation of HIV-1 replication. IL-1, IL-2, IL-3, IL-6, IL-12, granulocyte macrophage-colony stimulating factor (GM-CSF), and TNF-α/β have been identified as inducers of viral expression [11], prompting speculation that cytokines involved in the control and clearance of a *Plasmodium* infection, especially TNF-α, may cause the increase in HIV replication [5].

We developed a culture system to evaluate interactions between *P. falciparum* and HIV-1 *in vitro* using PBMCs collected from humans experimentally infected with *P. falciparum* under carefully controlled conditions in the context of a malaria human challenge trial (personal communication). Using this model system, we were able to further evaluate the mechanisms responsible for the deleterious interactions between HIV-1 and *P. falciparum,* and to determine whether these interactions are affected by prior exposure to *P. falciparum.*

## Results

### *P. falciparum*-infected Red Blood Cells (iRBCs) Stimulate More HIV-1 p24 Ag Production than uRBCs

**Figure 1:**
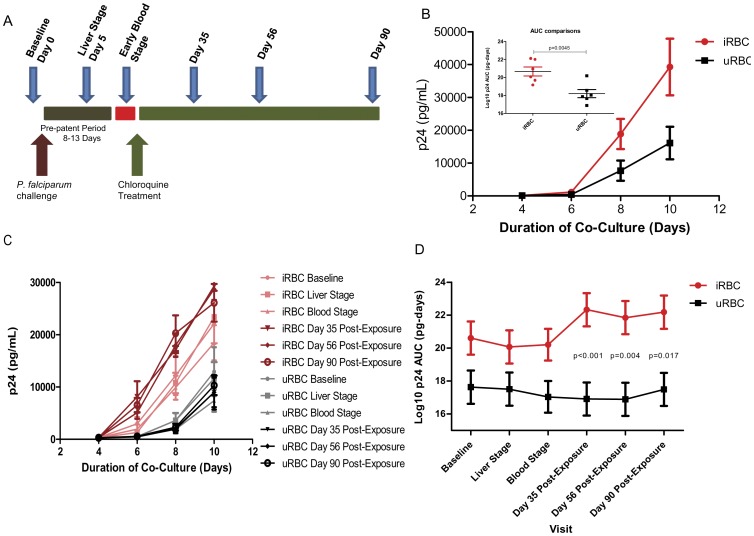
*Plasmodium falciparum* enhances HIV-1 production. A. Schematic representation of controlled *P. falciparum* infection. B. HIV production, as measured by p24 antigen concentration in the supernatants from PBMCs co-cultured with *P. falciparum* infected (iRBC, red line) or uninfected red blood cells (uRBC, black line). Inset shows area under the curve (AUC) comparisons of the amount of HIV p24 Ag produced in the iRBC versus uRBC co-cultures for 6 naïve donors averaged in the graph (Error bars represent SEM, p-value was determined with a paired two-tailed T-test). C. HIV production from PBMCs that were isolated at the 6 different visits from study participants and placed into co-culture with either iRBCs or uRBCs. Pink and black lines represent the means for the 5 participants (the blood stage time point included only 4 participants) at the three earliest time points (D0, D5, day of first parasitemia) and the burgundy and grey lines represent the means of the convalescent time points (∼D35, D56, D90), error bars represent the SEM. D. AUC comparisons of HIV production from the iRBC (red) and uRBC (black) co-cultures at all 6 visits. HIV production in the iRBC co-cultures is greater than the HIV production in the uRBC co-cultures at each visit; HIV produced in iRBC post-exposure co-cultures is greater than that produced in the iRBC co-cultures at the 3 early visits (p<0.001 day 35, p = 0.004 day 56, and p = 0.017 day 90 post-exposure). Each point is the mean for the 5 donors at that visit; the error bars represent the 95% confidence interval. p-values were determined using a repeated measures ANOVA, corrected for multiple comparisons.

Whole PBMCs isolated from HIV uninfected, malaria naïve donors produced significantly more HIV when co-cultured with iRBCs than when co-cultured with uRBCs (Figure 1B inset, p = 0.0045, area under the curve comparisons). The increase in HIV-1 production was evident by day 8 in culture. By day 10, HIV production in the iRBC co-cultures was increased about 2.5-fold over parallel co-cultures with uRBCs (Figure 1B).

### Repeat Exposure to *P. falciparum* Enhances Stimulation of HIV Production by iRBCs

To study the effects of acute *P. falciparum* infection on HIV-1 p24 Ag production by PBMCs after *in vitro* infection with HIV-1, we used PBMCs isolated from the controlled human malaria challenge participants. As noted above, all samples from any given study participant were thawed and studied in the same experiment. Sufficient PBMCs were available from 5 of the 6 malaria challenge trial participants for the baseline, liver stage, and later post-exposure time points and from 4 of the 5 participants at the blood stage time point. Once thawed and cultured overnight, the study design was identical to that described above. Figure 1C depicts HIV production in the iRBC/uRBC co-cultures for the 5 participants at the 6 different visits. For all of the participants at the baseline, liver stage, and blood stage visits, the amount of HIV produced from the PBMCs co-cultured with iRBCs (pink lines) was about 2 fold higher than the amount of HIV produced from the PBMCs co-cultured with uRBCs (grey lines). For all participants at the convalescent visits (day 35, 56, and 90 post malaria exposure time points), HIV production from the iRBC co-cultures (burgundy lines) was about 3 fold higher than that from the respective PBMCs co-cultured with uRBCs (black lines).

In order to further explore the relationship between *P. falciparum* stimulation and HIV-1 production by cells collected from malaria-exposed volunteers, we performed an area-under-the-curve calculation of p24 Ag production *in vitro* at each time point under each condition and evaluated serial differences for each patient. There was a significantly greater amount of HIV produced at each time point in the iRBC co-cultures compared to the uRBC co-cultures (p<0.001, Figure 1D). The amount of HIV produced from the uRBC co-cultures (black line) was unchanged across all the visits. In contrast, we observed a significant increase in HIV production when iRBCs were co-cultured with PBMCs collected at the day 35 (p<0.001), day 56 (p = 0.004), and day 90 (p = 0.017) post-exposure visits, compared to iRBCs co-cultured with PBMCs collected at earlier time points. Because the enhanced HIV production occurred after chloroquine-mediated clearance of parasitemia in the study participants, our results suggest that development of malaria specific cellular immunity, rather than activated effector cells, are responsible for the heightened HIV production. In order to determine if CD4+ memory T-cells were indeed responsible for the increase we saw in HIV production, we compared surface activation markers on total CD4+ T-cells, memory (CD45RO+) CD4+ T-cells, as well as total CD8+ T-cells and memory (CD45RO+) CD8+ T-cells in PBMCs obtained on Day 56 post-malaria exposure compared to surface activation markers expressed by these cell types when obtained from malaria naïve donors. Limitations in the number of available baseline PBMC samples from the malaria exposed participants precluded a matched longitudinal study to examine differences in memory cell activation to malaria antigens. In these experiments, cells were analyzed at 48, 72, and 96 hours post co-culture with either iRBCs or uRBCs for surface expression of cellular activation markers (Figure S1). We observed a general trend toward increased CD4 memory T-cell activation in the Day 56 malaria exposed donor PBMCs compared to naïve control PBMCs after 72 and 96 hours (Figure S1B). Statistical significance was noted with the CD25 (at 72 hours, p = 0.046), CD69 (at 48 hours, p = 0.008; at 72 hours, p = 0.034) and CD38 (at 72 hours, p = 0.011; at 96 hours, p = 0.003) markers but not with the HLA-DR marker or among HLA-DR/CD38 double positive cells. Within the total CD4 cell population, we noted only upregulation of CD25 (p = 0.018) and CD69 (p = 0.027) in the *P. falciparum* exposed donor PBMCs compared to naïve control PBMCs at the 72 hour time point (Figure S1A). There were not sufficient PBMCs from the malaria challenge trial participants to assess T-cell activation in cells cultured with media alone. In the cells from the malaria naïve donors, we saw no difference in the amount of CD4 or CD8 activation for any of the markers in the media alone condition compared to the uRBC condition (data not shown).

### Increase in TNF-α, IFN-γ, and MIP-1α Secretion is Enhanced in iRBC Co-cultures Following in vivo Infection with *P. falciparum*

**Figure 2:**
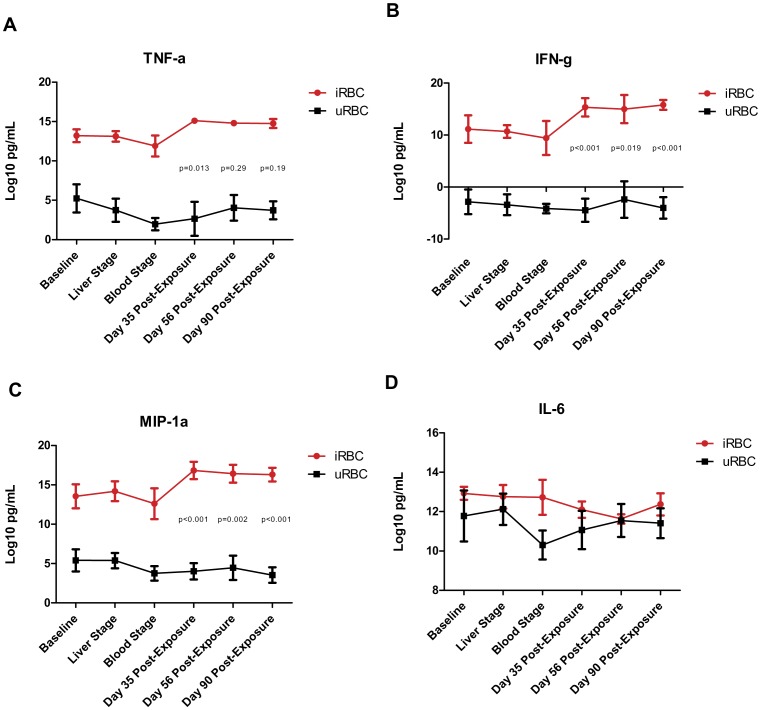
*Plasmodium falciparum* stimulates enhanced secretion of TNF-α, IFN-γ, and MIP-1α, but not IL-6. PBMCs from the malaria challenge trial participants were infected with HIV and co-cultured with iRBC and uRBC at the 6 visits as described earlier. Cytokine secretion in the iRBC (red lines) and uRBC (black lines) co-culture supernatants was measured using the BioPlex platform. Cytokine production was measured at days 1, 4, 6, and 8 post initiation of co-culture and the area under the curve was calculated for each participant at each visit and plotted in A – D as the average for the 5 participants, error bars represent the 95% CI. Repeated measures ANOVA was used to determine significance in the difference in amount of cytokines secreted (iRBC-uRBC) at the post-exposure time points compared to the difference in amount of cytokine secreted (iRBC-uRBC) at baseline. p-values have been corrected for multiple comparisons: TNF-α day 35 p = 0.013, day 56 p = 0.29, day 90 p = 0.19; IFN-γ day 35 p<0.001, day 56 p = 0.019, day 90 p<0.001; MIP-1α day 35 p<0.001, day 56 p = 0.002, day 90 p<0.001. There was an increase in TNF-α, IFN-γ, and MIP-1α (A–C) secretion in the iRBC co-cultures compared to the uRBC co-cultures at all time points and an enhanced secretion at the convalescent time points. There was no difference in IL-6 secretion (D).

We then measured cytokine production in culture supernatants to examine relationships between HIV production and cytokine secretion. We measured the secretion of 7 different cytokines: IL-4, IL-6, IL-10, IL-17, TNF-α, IFN-γ, and MIP-1α. The levels of IL-4, IL-10, and IL-17 secretion were below the limit of detection of the BioPlex assay (data not shown). Increases in the secretion of TNF-α, IFN-γ, and MIP-1α were observed in the co-cultures that had been established with iRBCs compared to uRBCs. Production of these cytokines was further increased in PBMCs obtained 35, 56, and 90 days post malaria challenge compared to those obtained at baseline or at early stages of *P. falciparum* infection (Figure 2A–C, TNF-α: day 35 p = 0.013, day 56 p = 0.29, day 90 p = 0.19; IFN-γ: day 35 p<0.001, day 56 p = 0.019, day 90 p<0.001; MIP-1α: day 35 p<0.001, day 56 p = 0.002, day 90 p<0.001). These cytokine profiles closely mirror the HIV production profiles in Figure1D. IL-6 secretion was similar in iRBC and uRBC co-cultures (Figure 2D).

### Systemic Inflammation does not Correlate to Increased HIV Production in the Co-cultures

**Figure 3:**
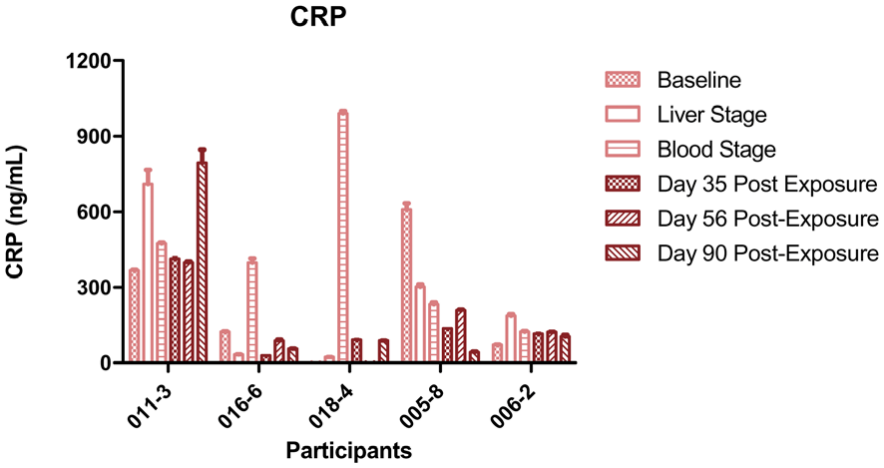
General inflammation in the periphery does not correlate to HIV production in co-cultures. Generalized immunological activation as assessed by C-Reactive Protein (CRP) levels in the plasma collected at the time of PBMC isolation in the five malaria challenge participants. CRP was detected by ELISA. The light pink bars correspond to the early visits (baseline, liver stage, time of first parasitemia) and the burgundy bars correspond to the three post-exposure time points (day 35, day 56, and day 90). The bars show the average CRP levels in the plasma at the time of PBMCs isolation for the 5 participants. Error bars represent the SEM for the triplicate wells run at each time point. Levels of CRP in the plasma did not increase at the post-exposure time points when the increase in HIV production was observed, suggesting that the increase in HIV production was specific to malaria antigen re-exposure and not a result of generalized immune activation.

In order to evaluate whether increases in HIV production at the later time points might be related to generalized immune activation following recovery from acute *P. falciparum*, we measured levels of C-Reactive Protein (CRP) in plasma obtained at the time of PBMC isolation. For all but one participant (011-3), levels of CRP appear to be higher during the early time points than at the later time points (Figure 3). Thus, CRP levels were generally higher during active infection compared to convalescent samples, where HIV-1 was seen to increase. In fact, a spike in the CRP levels was noted in two participants (018-4 and 016-6) at the blood stage time point of the active malaria infection, but there was no corresponding increase in HIV production at the same time point. This supports the concept that increased HIV production during the convalescent phase is not the result of systemic inflammation.

## Discussion

Using PBMCs and plasma collected from donors before, during and after controlled infection with *P. falciparum*, we explored interactions between HIV-1 and *P. falciparum*. We confirm that iRBCs co-cultured with PBMCs from malaria naïve donors enhance replication of HIV-1, and show that HIV-1 production is further enhanced in PBMCs collected in the convalescent period after experimental infection with *P. falciparum*. HIV-1 replication after exposure to *P. falciparum* infected erythrocytes was accompanied by increased secretion of pro-inflammatory cytokines TNF-α, IFN-γ, and MIP-1α, which was also enhanced after experimental *P. falciparum* infection. In addition, we noted a trend toward increased memory CD4+ T-cell activation in response to iRBCs during the convalescent period. Enhanced HIV production *in vitro* following controlled human infection with *P. falciparum* was not associated with systemic inflammation *in vivo* as assessed by plasma C-reactive protein levels.

Under the controlled conditions of the standard human challenge model [12]–[14], subjects are diagnosed and treated at low parasite density, often prior to the onset of clinical symptoms and likely prior to the development of the cytokine storm observed in severe bouts of clinical malaria in non-immune individuals [15]. Despite the limited duration of parasitemia in the experimental malaria infection, production of HIV-1 by *P. falciparum-*exposed PBMCs was substantially enhanced following treatment and resolution of the blood stage of infection. Although not examined in these initial experiments, the changes we observed might have been more pronounced had study participants progressed to clinical disease. Our data suggests that T-cell activation in these participants was modest at the time parasitemia was first demonstrated on peripheral blood smears, although flow cytometry was not performed longitudinally to assess levels of activation of unstimulated T-cells before and after experimental infection. It has previously been shown that chloroquine reduces the number of activated CD8+ memory T-cells and, to a lesser extent, CD4+ memory T-cells in the peripheral blood and inhibits the production and secretion of TNF-α [16], [17]. Since the participants in the malaria challenge trial received chloroquine after the blood stage blood draw, PBMCs isolated at the first three time points were free of the immunomodulatory effects of chloroquine. PBMCs collected at the later post-exposure time points (days 35, 56, and 90) might have been exposed to varying levels of chloroquine (in view of its biological half-life of 1–2 months). Thus, although chloroquine may have immunomodulatory effects, the time points at which we noted enhancement of HIV-1 production were those when it might have been present and we would have expected immunomodulatory effects of chloroquine to exert an effect opposite to that which we observed.

The role of cytokines during malaria has been extensively studied. In controlled *Plasmodium* infections of malaria-naïve subjects, serum levels of pro-inflammatory cytokines, including TNF-α, IL-6, IFN-γ, and IL-12p40, increase at the time that parasites emerge from the liver and at the first appearance of parasitized erythrocytes [10]. McCall *et al.* reported that PBMCs isolated from participants who were infected with *P. falciparum* in a controlled human malaria infection and exposed to cryopreserved iRBCs secreted higher levels of IFN-γ by NK cells than naïve PBMCs, and that this response was dependent on CD4 help [18]. In semi-immune individuals living in malaria endemic areas, the amount of IFN-γ secreted is lower upon repeat exposure to malaria antigens than in malaria naïve individuals, and malaria episodes are less severe [19]. Separately, several TNF-α alleles have been correlated to increased plasma TNF levels, increased susceptibility to severe malaria [20], and increased susceptibility to cerebral malaria [21]. Higher levels of circulating TNF-α were found in adults and children with severe malaria compared to both uncomplicated malaria cases and healthy individuals [20], [22]. While IFN-γ is a pro-inflammatory cytokine, it inhibits HIV infection *in vitro* and has been used in patients with advanced AIDS to reduce the number of opportunistic infections [23], [24]. Thus, even though both TNF-α and IFN-γ are classified as pro-inflammatory cytokines and even though both increase during acute bouts of clinical malaria, TNF-α is a better candidate cytokine than IFN-γ to stimulate HIV production during *P. falciparum* infection. Furthermore, peak plasma levels of TNF-α do not decrease with repeat *P. falciparum* infections while those of IFN-γ decline with repeated infection.

Our data extend those of Xiao *et al.,* who employed a similar *in vitro* system to examine the effects of *P. falciparum* on HIV-1 production by CD8+ T-cell depleted PBMCs from malaria-naïve individuals [25]. In their hands exposure to *P. falciparum* merozoites and hemozoin increased HIV-1 replication [25]. In our studies, in an effort to more closely mimic a natural malarial infection, we stimulated unfractionated PBMCs with *P. falciparum*-infected red blood cells rather than with merozoites or hemozoin. In the model system reported by Xiao, HIV-1 replication was the result of increased TNF-α production, but not the result of IL-6 production, demonstrated by the use of TNF-α and IL-6 blocking antibodies. We also observed that TNF-α increased in iRBC co-cultures compared to uRBC co-cultures and in post-exposure iRBC cultures compared to the early cultures, while IL-6 production did not change significantly in any of these conditions. Although there are clear-cut differences between the experimental systems and although neither system fully captures all aspects of natural infection, HIV-1 production was enhanced by *P. falciparum* exposure under both sets of experimental conditions. It would be of significant interest to further examine the cell types and mechanisms driving the increase in HIV production in response to *P. falciparum* stimulation.

Memory CD4+ T-cells have been an active area of inquiry in the malaria field. Some have proposed that memory responses to malaria are very short lived and that malaria specific CD4+ T-cells are deleted, resulting in very little memory against future infection [26], [27]. Others have proposed that the apoptosis of antigen-specific CD4 cells would be expected following resolution of acute infection [28]. Animal models of malaria have shown that fully functional memory CD4+ T-cells are maintained for prolonged periods of time [29]. Recently, Wipasa *et al.* showed that malaria specific CD4+ effector memory responses decay with a half-life of about 3 years and that malaria specific CD4+ central memory responses are maintained for at least six years after the last documented clinical episode of malaria in humans [30]. If the enhanced production of HIV-1 observed in our system is truly attributable to the development of adaptive immune responses to *P. falciparum* antigens, our results suggest that memory CD4+ T-cells also develop in association with subclinical malarial infection and that they persist for at least 90 days post-exposure.

Our study examined for the first time HIV-1 replication (*in vitro)* induced by exposure to *P. falciparum* using samples derived from human volunteers experimentally infected with malaria. As others have demonstrated, malarial antigens increase HIV-1 replication *in vitro* prior to experience with the parasite *in vivo.* We have demonstrated that this increase is further enhanced following an experimental infection with *P. falciparum.* These findings provide experimental support to the clinical observations that plasma levels of HIV-1 RNA rise during clinical bouts of malaria [3]–[5]. Although our study examined an initial exposure to *P. falciparum,* persons living in regions endemic for *P. falciparum* undergo repeated exposures to the parasite and serial bouts of clinical illness. In the course of these repeated infections, *P. falciparum-*specific CD4+ T-cells develop and play a critical role in ameliorating morbidity and mortality [30]–[32]. In the setting of HIV-1 infection it might be expected that pathogen specific CD4+ cells would be activated by malarial infection and, thus, would be likely to be especially vulnerable to HIV-1 infection. Such an interaction would be expected both to erode malaria-specific immunity and to contribute to enhanced replication of HIV-1 as has been demonstrated both in the case of HIV-1 and *M. tuberculosis*-specific immunity [33]. This unfortunate immunopathogenic interaction would then form, at least in part, the basis for a progressively more deleterious bidirectional clinical interaction between these two pathogens with advancing HIV-1 associated immunodeficiency.

Further studies are needed to more fully examine the interactions between HIV-1 and *P. falciparum* infection both in the clinic and in *in vitro* model systems. We believe that our studies lend further support to the emerging evidence that these pathogens are not indifferent to each other in areas where both pathogens are endemic. This experimental culture system provides a convenient platform in which to more carefully examine the bidirectional interactions between these pathogens both in patients with recurrent bouts of clinical malaria and in those with a subclinical infection.

## Materials and Methods

### PBMC Collection and Isolation

PBMCs were isolated from human subjects enrolled in an experimental malaria challenge trial (protocol MC-001) at the Malaria Clinical Trials Center at Seattle Biomedical Research Institute (personal communication). The experimental infection of human subjects was conducted according to standard procedures as previously described [34]. Briefly, healthy malaria-naïve adult volunteers were infected with *Plasmodium falciparum* sporozoites from bites of five *P. falciparum* (strain NF54)-infected *A. stephensi* mosquitoes under controlled conditions. Volunteers were closely monitored in the post-challenge period and treated with standard doses of chloroquine phosphate (CQ) upon diagnosis of parasitemia by positive thick blood films. Blood sampling for isolation of (PBMCs occurred prior to challenge (baseline) and at regular intervals after challenge (Figure 1A) including day 5 (corresponding to the liver stage of parasite development), the day of the first positive blood smear (corresponding to the blood stage), and in the post-treatment period approximately 35, 56, and 90 days following mosquito bites. Cryopreserved PBMCs were thus available prior to the challenge, during the liver and blood stage parasitemia, and at three time points during convalescence.

PBMCs were isolated and cryopreserved according to standard methods and frozen within 8 hours of venipuncture to ensure optimal viability [35] under Good Clinical Laboratory Practices (GCLP). Fresh PBMCs were isolated by Histopaque 1077 centrifugation and cultured overnight at 37°C in 5% CO_2_ in IL-2/PHA free media (10% FBS, 1% Pen-Strep in RPMI 1640) and used the following day in co-cultures. Frozen PBMCs were isolated at each designated collection point and cryopreserved in 10% DMSO/90% FBS and stored at −80 C until use in the designated assay. Fresh PBMCs were obtained from malaria naïve donors enrolled in a separate protocol (HS103) at Seattle Biomedical Research Institute. Both protocols were approved by the Western Institutional Review Board.

### Co-cultures

On the day of study, PBMCs from all time points from a given volunteer were thawed and placed in R10 media overnight (10% FBS, 1% Pen-Strep, 1% L-Glutamine, RPMI 1640). The day following the thaw, PBMCs were placed in 96-well plates (2×10^5^ cells/well/200 µL) and infected with HIV-1 (San Diego field isolate S-144; MOI = 25) without exogenous mitogens or cytokines in R20 media (20% FBS, 1% Pen-Strep). Red blood cells (either *P. falciparum* infected or uninfected) were then added to selected wells in a 10∶1 ratio of RBCs to PBMCs (2×10^6^ RBCs/well/200 µL). All conditions were run in triplicate. After 22 hours, the entire 200 µL of media was collected and replaced with new media to eliminate excess virus. 100 µL of the culture supernatants was collected at days 4, 6, 8 and 10 and replaced with fresh medium. These supernatants were frozen at −80°C and used to determine HIV p24Ag and cytokine levels. Viral production was quantified by determining amount of p24 antigen in the culture supernatants by p24 Antigen capture ELISA (Perkin Elmer) by the UCSD CFAR Translational Virology Core.

### *Plasmodium Falciparum* Culture

*P. falciparum* NF54 parasites were grown in type O human RBCs (obtained from malaria naïve donors enrolled in blood draw program (protocol HS103) at Seattle Biomedical Research Institute) in RPMI 1640 (Invitrogen) with 5 g/L albumax (Invitrogen), 2 g/L dextrose (Fisher), 50 mg/L hypoxanthine (Sigma), 2.25 g/L sodium bicarbonate (Sigma), 11 mg/L gentamycin (Invitrogen), and 5% pooled human AB serum (Valley Biomedical). Parasite chambers were gassed with 5% O_2_/5% CO2/90% N_2_ and incubated at 37°C. Parasite cultures were maintained continuously and split 1–2 days prior to setting up co-cultures, uRBCs were also placed into culture on the same day as iRBCs were split. Unwashed iRBCs were used once 6–7% of the RBCs in the culture were parasitized as assessed by light microscopy. *P. falciparum* stage was not synchronized. Cultures were routinely monitored for mycoplasma contamination by PCR (Takara) and shown to be mycoplasma free.

### Cytokine Quantification

Cytokine levels were measured in culture supernatants using a BioPlex platform (BioRad). A 6-plex kit containing IL-10, IL-17, IL-6, TNF-α, IFN-γ and MIP-1α or a 5-plex kit containing IL-4, IL-6, TNF-α, IFN-γ and MIP-1α was used according to the manufacturer’s protocol. Briefly, the antibody coupled beads were mixed with 50 µL of the collected supernatants, which were diluted 1∶2 with media and incubated on a shaker for 30 minutes. After three washes, the detection antibodies were added and the plate was incubated on the shaker for 30 minutes. After another three washes, Streptavidin-PE was added to each well and plates were incubated on the shaker for 10 minutes. The beads were resuspended in 1% formaldehyde in assay buffer and after a 30 second shake the plate was read on a Bioplex200 (BioRad). An 8-point standard curve was used to determine cytokine concentrations using a 5 parameter logistic regression curve. Detection limits for cytokines are as follows: IL-4, 0.7 pg/mL; IL-6, 2.6 pg/mL; IL-10, 0.3 pg/mL; IL-17, 3.3 pg/mL; IFN-γ, 6.4 pg/mL; TNF-α, 6.0 pg/mL; MIP-1α, 1.6 pg/mL.

### CRP ELISA

CRP Quantikine Kit (R&D) was used to quantify the CRP in the plasma of the participants. Briefly, plasma (diluted 1∶100) was added to a 96 well plate pre-coated with anti-human CRP antibodies and incubated at room temperature for 2 hours. The plate was washed 4 times with wash buffer. A horseradish peroxidase-conjugated CRP antibody was added and incubated at room temperature for 2 hours. Followed by another 4 washes with wash buffer, color solutions A and B were added in equal amounts and were allowed to incubate at room temperature for 30 minutes at which point a stop solution was added. The plate was read at 450 nm and a plate correction was read at 540 nm. The log_10_ of the concentration for the standards was plotted against the log_10_ of the respective OD values and a regression line was obtained that was used to calculate the CRP concentrations for the obtained OD values for the unknowns.

### Flow Cytometry

PBMCs were collected, frozen, and then thawed as described above. PBMCs from day 56 post malaria challenge and from malaria naïve donors were plated (2×10∧5 PBMCs/well) in a 96 well plate and co-cultured with either iRBCs or uRBCs (2×10∧6 RBCs/well) in triplicate. Cultures were incubated at 37°C, 95% O_2_ for 48, 72, or 96 hours. At the indicated time point, all three wells for a given condition were combined into a single FACS tube. Cells were washed once in FACS buffer (PBS +2% FBS), resuspended in 50 µL of Live/dead – Aqua stain (Invitrogen) and stained with either CD3-FITC (eBioscience), CD4-Pacific Blue (eBioscience), CD8-APC (BD Pharmingen), CD45RO-PE (BD Pharmingen), HLA-DR-PerCP (Biolegend), and CD38-PE-Cy5.5 (BD Pharmingen) or CD3-FITC, CD4-Pacific Blue, CD8-APC, CD45RO-PE, CD69-PerCP-Cy7 (eBioscience), and CD25-PE-Cy5.5 (eBioscience). Cells were stained at room temperature for 20 minutes and washed twice with FACS buffer. The red blood cells were then lysed using 120 µL of BD FacsLyse at room temperature for 15 minutes, washed once with PBS and resuspended in 130 µL of 2% formaldehyde in distilled H_2_O. Samples were subjected to flow cytometric analysis within 18 hours of fixing on the BD LSRII. All data were analyzed using FlowJo (Treestar).

### Statistical Methods

Total HIV-1 p24Ag and cytokine production were calculated over the 10 days in culture using a trapezoidal method (area under the curve). Log-transformed HIV p24Ag and cytokine production were compared between the iRBC and uRBC groups at each time point following HIV infection of co-cultures to which iRBCs or uRBCs had been added, and between earlier and later time points following exposure, using a subject-specific random intercepts ANOVA model including time (as a factor), group (iRBC versus uRBC), their interaction, and a random subject effect. Therefore, different mean values were allowed at each time point and for each of the two groups for the log_10_ p24 AUC values, while correlation of data points measured for the same subject was modeled by a normally-distributed random intercept. The main comparisons of interest between the iRBC and uRBC groups were of the change in mean log_10_ p24 AUC between baseline and later stages. These were evaluated by the Wald test, with a Bonferonni correction for multiple comparisons. Graphs were made in GraphPad Prism Software.

### Ethics Statement

Human studies undertaken at the Seattle Biomedical Research Institute were reviewed and approved by the Western Institutional Review Board. Studies undertaken at UCSD were reviewed and approved by the UCSD Human Research Protections Program. Study participants provided written informed consent prior to their participation in the study.

## Supporting Information

Figure S1 **Activation of memory and total CD4/8 T-cells by iRBCs in D56 post malaria challenge PBMCs.** PBMCS were cultured with iRBCs or uRBCs (without HIV) for 48, 72 or 96 hours. Cells were stained with either CD3-FITC, CD4-Pacific Blue, CD8-APC, CD45RO-PE, HLA-DR-PerCP, and CD38-PE-Cy5.5 or CD3-FITC, CD4-Pacific Blue, CD8-APC, CD45RO-PE, CD69-PerCP-Cy7, and CD25-PE-Cy5.5 and acquired using an LSRII. The percent of cells activated by iRBCs was normalized to the amount of activation due to uRBCs; this value was then logged and plotted. Line and error bars represent the mean and standard error of the mean. All values above the dotted line represent stimulation due to iRBCs. p-values were determined using an unpaired, two-tailed T-test. A. CD4 cells from the D56 post-malaria exposure PBMCs only show increased expression of CD25 (p = 0.018) and CD69 (p = 0.027) at 72 hours and there are no obvious trends toward increased activation in the malaria exposed PBMCs. B. While expression of CD 69 at 48 hours (p = 0.008), CD38 (p = 0.011), CD25 (p = 0.046), and CD69 (p = 0.034) at 72 hours and CD38 (p = 0.003) at 96 hours are significantly increased in CD4+CD45RO+ D56 post malaria challenge PBMCs compared to naïve controls at 72 hours, there is also a trend toward increased activation in the HLA-DR/CD38 double positive cells in the PBMCs from malaria exposed donors compared to naïve controls at both 72 and 96 hours post co-culture in the memory CD4 compartment. C – D. For the total and memory CD8 T-cells, there is increased activation in the malaria exposed Day 56 PBMCs at 72 and 96 hours compared to naïve controls. For total CD8 cells at 72 hours, there is increased expression of CD38 (p = 0.016) and CD69 (p = 0.001); at 96 hours, there is increased expression of HLA-DR/CD38 double positives (p = 0.025) and CD38 alone (p = 0.015). For memory CD8 cells at 72 hours, there is increased expression of CD38 (0.036) and CD69 (0.001); at 96 hours, there is increased expression of CD38 alone (p = 0.0003).
(TIF)
