# Supplementary figures and images for: P. falciparum Enhances HIV Replication in an Experimental Malaria Challenge System
Source: PLoS One. 2012 Jun 26;7(6):e39000. doi: 10.1371/journal.pone.0039000 (PMC3383717; doi:10.1371/journal.pone.0039000)

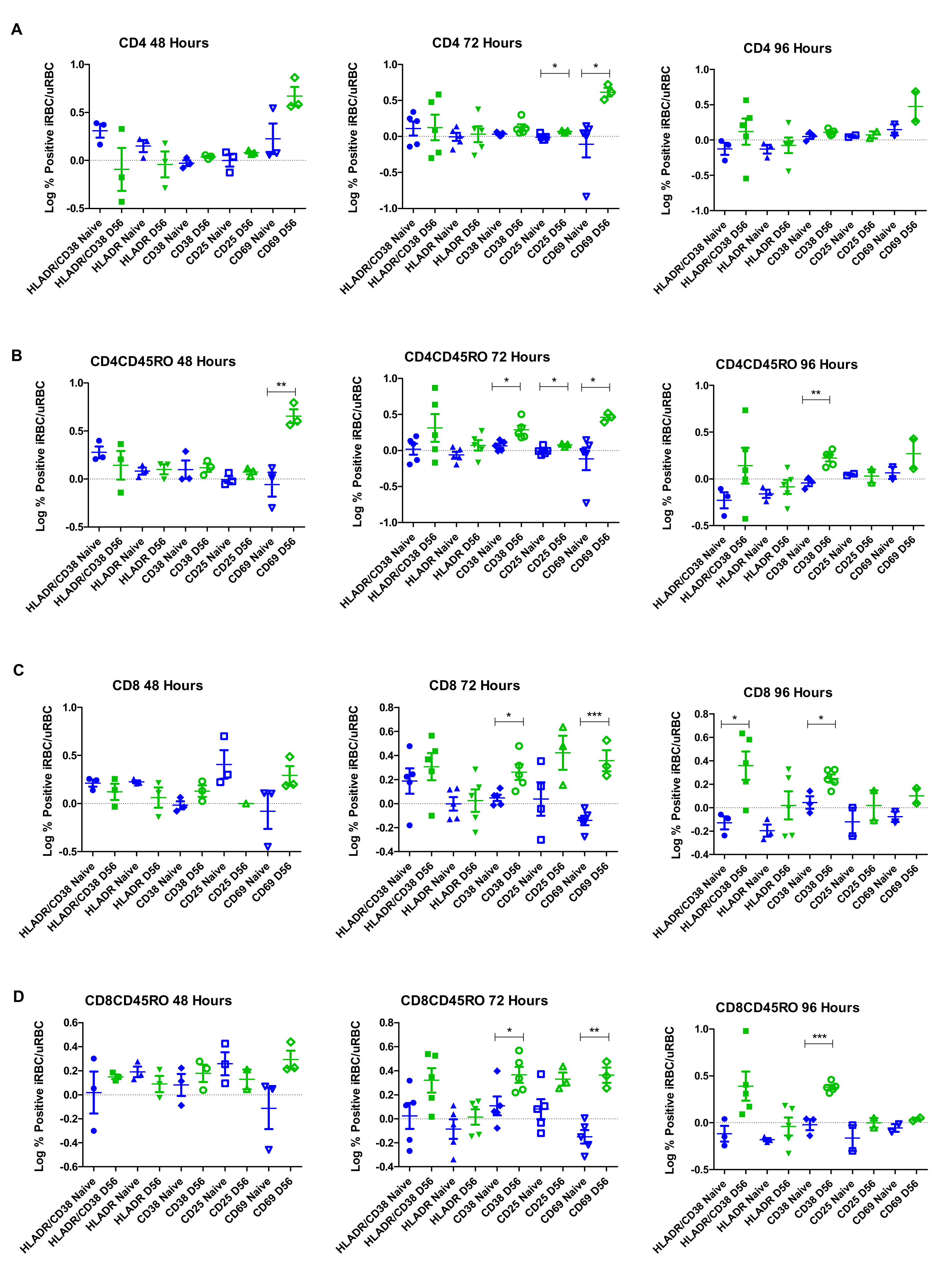

Supplement: Figure S1 — Activation of memory and total CD4/8 T-cells by iRBCs in D56 post malaria challenge PBMCs. PBMCS were cultured with iRBCs or uRBCs (without HIV) for 48, 72 or 96 hours. Cells were stained with either CD3-FITC, CD4-Pacific Blue, CD8-APC, CD45RO-PE, HLA-DR-PerCP, and CD38-PE-Cy5.5 or CD3-FITC, CD4-Pacific Blue, CD8-APC, CD45RO-PE, CD69-PerCP-Cy7, and CD25-PE-Cy5.5 and acquired using an LSRII. The percent of cells activated by iRBCs was normalized to the amount of activation due to uRBCs; this value was then logged and plotted. Line and error bars represent the mean and standard error of the mean. All values above the dotted line represent stimulation due to iRBCs. p-values were determined using an unpaired, two-tailed T-test. A. CD4 cells from the D56 post-malaria exposure PBMCs only show increased expression of CD25 (p = 0.018) and CD69 (p = 0.027) at 72 hours and there are no obvious trends toward increased activation in the malaria exposed PBMCs. B. While expression of CD 69 at 48 hours (p = 0.008), CD38 (p = 0.011), CD25 (p = 0.046), and CD69 (p = 0.034) at 72 hours and CD38 (p = 0.003) at 96 hours are significantly increased in CD4+CD45RO+ D56 post malaria challenge PBMCs compared to naïve controls at 72 hours, there is also a trend toward increased activation in the HLA-DR/CD38 double positive cells in the PBMCs from malaria exposed donors compared to naïve controls at both 72 and 96 hours post co-culture in the memory CD4 compartment. C – D. For the total and memory CD8 T-cells, there is increased activation in the malaria exposed Day 56 PBMCs at 72 and 96 hours compared to naïve controls. For total CD8 cells at 72 hours, there is increased expression of CD38 (p = 0.016) and CD69 (p = 0.001); at 96 hours, there is increased expression of HLA-DR/CD38 double positives (p = 0.025) and CD38 alone (p = 0.015). For memory CD8 cells at 72 hours, there is increased expression of CD38 (0.036) and CD69 (0.001); at 96 hours, there is increased expression of CD38 alone (p [file pone.0039000.s001.tif]
